# Salt-sensitive hypertension in GR mutant rats is associated with altered plasma polyunsaturated fatty acid levels and aortic vascular reactivity

**DOI:** 10.1007/s00424-024-03014-y

**Published:** 2024-09-10

**Authors:** S. Verouti, G. Aeschlimann, Q. Wang, D. Ancin Del Olmo, A. C. Peyter, S. Menétrey, D. V. Winter, A. Odermatt, D. Pearce, E. Hummler, P. E. Vanderriele

**Affiliations:** 1 Department of Biomedical Sciences, University of Lausanne, Lausanne, Switzerland; 2 National Center of Competence in Research, Kidney.CH, Lausanne, Switzerland; 3 Department for BioMedical Research (DBMR), University of Bern, Bern, Switzerland; 4 Division of Nephrology and Hypertension, Lausanne University Hospital (CHUV), Lausanne, Switzerland; 5 Neonatal Research Laboratory, Clinic of Neonatology, Department Woman-Mother-Child, Lausanne University Hospital (CHUV) and University of Lausanne, Lausanne, Switzerland; 6 Division of Molecular and Systems Toxicology, Department of Pharmaceutical Sciences, University of Basel, Basel, Switzerland; 7 Department of Medicine and Cellular & Molecular Pharmacology, University of California, San Francisco, USA

**Keywords:** Adrenal gland hyperplasia, Hypertension, Glucocorticoid receptor, Soluble epoxide hydrolase, Chrousos syndrome

## Abstract

In humans, glucocorticoid resistance is attributed to mutations in the glucocorticoid receptor (GR). Most of these mutations result in decreased ligand binding, transactivation, and/or translocation, albeit with normal protein abundances. However, there is no clear genotype‒phenotype relationship between the severity or age at disease presentation and the degree of functional loss of the receptor. Previously, we documented that a GR^+/−^ rat line developed clinical features of glucocorticoid resistance, namely, hypercortisolemia, adrenal hyperplasia, and salt-sensitive hypertension. In this study, we analyzed the GR^+/em4^ rat model heterozygously mutant for the deletion of exon 3, which encompasses the second zinc finger, including the domains of DNA binding, dimerization, and nuclear localization signals. On a standard diet, mutant rats exhibited a trend toward increased corticosterone levels and a normal systolic blood pressure and heart rate but presented with adrenal hyperplasia. They exhibited increased adrenal soluble epoxide hydroxylase (sEH), favoring an increase in less active polyunsaturated fatty acids. Indeed, a significant increase in nonactive omega-3 and omega-6 polyunsaturated fatty acids, such as 5(6)-DiHETrE or 9(10)-DiHOME, was observed with advanced age (10 versus 5 weeks old) and following a switch to a high-salt diet accompanied by salt-sensitive hypertension. In thoracic aortas, a reduced soluble epoxide hydrolase (sEH) protein abundance resulted in altered vascular reactivity upon a standard diet, which was blunted upon a high-salt diet. In conclusion, mutations in the GR affecting the ligand-binding domain as well as the dimerization domain resulted in deregulated GR signaling, favoring salt-sensitive hypertension in the absence of obvious mineralocorticoid excess.

**Supplementary Information:**

The online version contains supplementary material available at 10.1007/s00424-024-03014-y.

## Introduction

Hypertension is the leading cause of cardiovascular disease and one of the major causes of premature death worldwide [30]. Its development is influenced by several factors, including genetic background, obesity, and excess salt intake [34] ranging from 8 to 10 g daily [2]. Salt-sensitive hypertension is a common type of high blood pressure that is exacerbated by a high-salt diet and affects approximately 30% of healthy humans [2] and 50% of individuals with hypertension [41]. Several pathways adjust salt excretion to match changes in dietary salt intake in the kidney. Gitelman patients show renal salt wasting due to inactivating mutations of the SLC12A3 gene encoding the thiazide-sensitive sodium chloride co-transporter NCC, whereas Liddle patients retain renal sodium due to mutation in the SCNN1β and SCNN1γ genes encoding the b- and g-subunit of ENaC [3, 37]. Furthermore, an implication of the vascular, the sympathetic nervous, the gastrointestinal, and the immune system and the skin was demonstrated as well [10]. Evidence of the heritability of salt sensitivity was documented, and allelic variants of candidate genes not only affect the renal sodium transport like the angiotensin II type 1 receptor, the 11β-hydroxysteroid dehydrogenase (11βHSD), or the chloride voltage-gated channel Ka (CLCNKA) but also vascular reactivity like the solute carrier family 24 member 3 (SLC24A3) or the endothelin receptor type B (ENDRB) (for review, see [32]). Several rodent models were used to study salt-sensitive hypertension like Dahl salt-sensitive rats, DOCA-salt-induced mice, or genetically engineered mice mutant for the 11βHSD2 or the βENaC subunit genes (for review, see [7, 32]). Additionally, sex-specific salt sensitivity was reported following high-salt diet in female Balb/c mice resulting in impaired endothelium-dependent vasodilation [12] and in male C57Bl/6J mice exhibiting sympathetic overactivity without renal sodium retention [47]. Following norepinephrine or isoproterenol treatment, C57Bl/6J mice exhibited NCC-mediated sodium retention and salt-sensitive hypertension. In these mice, increased glucocorticoid receptor binding was due to b-adrenergic receptor stimulation suggesting a role for the development of salt-induced hypertension [36].

**Fig. 1:**
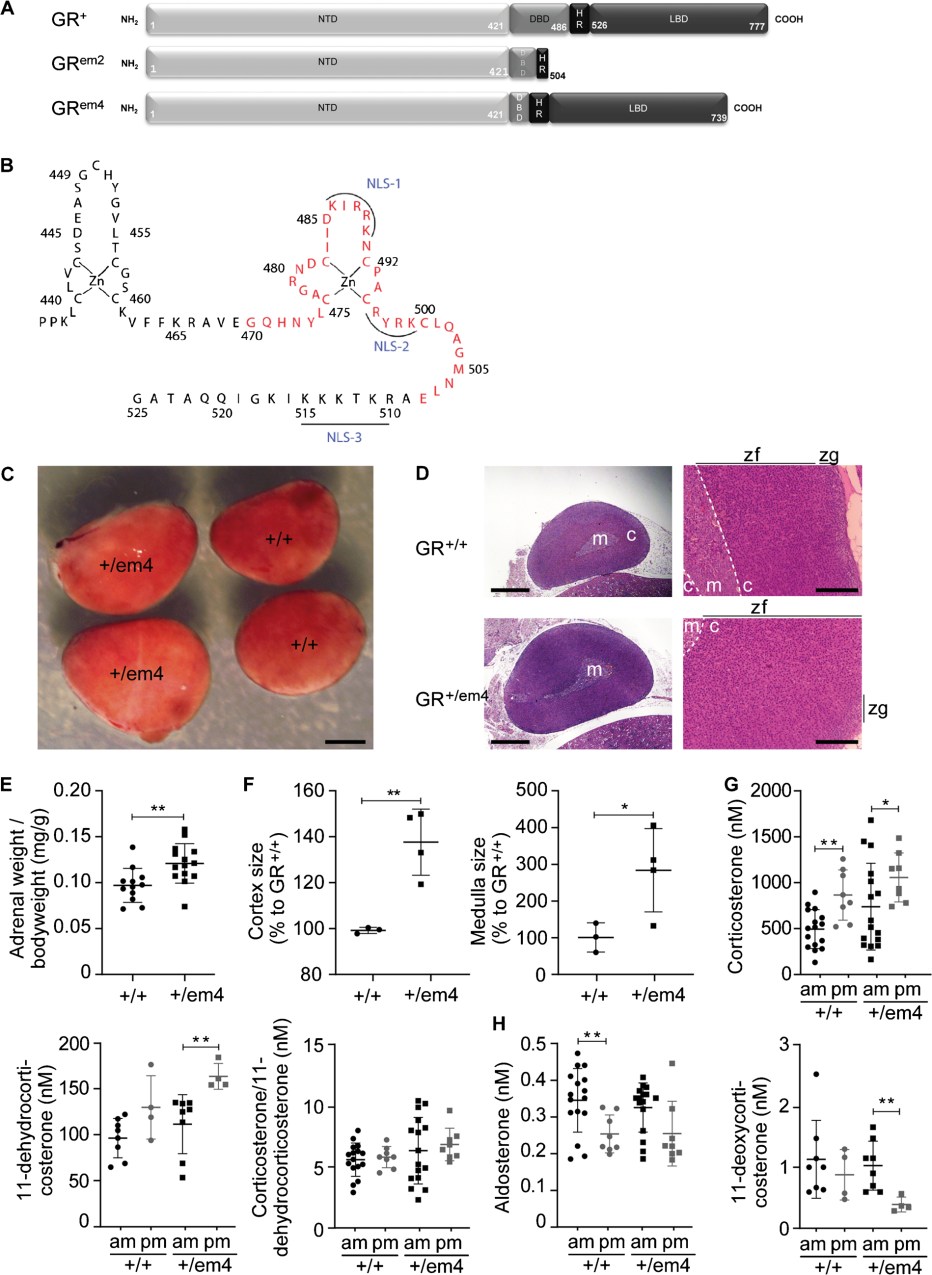
GR^+/em4^ rats presented adrenal hyperplasia of the cortex and the medulla accompanied by a trend toward an increase in the plasma glucocorticoid concentration under standard diet. **A** Scheme of the wild-type (GR^+^, upper panel) and the mutated GR^em2^ (middle) and GR^em4^ (lower panel) structure of the GR. **B** Representation of the zinc finger domain of the GR with the deleted amino acids in red. **C** Representative macroscopic images (scale bar, 1 mm) and **D** hematoxylin/eosin-stained sections of whole adrenal glands (left panels; scale bar, 1 mm) and cortex (right panels; scale bar, 300 µm) from 3- to 4-week-old male GR^+/+^ and GR^+/em4^ rats (*n* = 3) fed a standard salt diet; zf, zona fasciculata; zg, zona glomerulosa. **E** Measurement of the adrenal weight/body weight ratio (GR^+/+^, *n* = 12; GR^+/em4^, *n* = 14) and **F** cortex (left) and medulla (right panel) size (GR^+/+^ (*n* = 3) and GR^+/em4^ (*n* = 4)). **G** Determination of plasma concentrations of the glucocorticoids corticosterone, 11-dehydrocorticosterone and the corticosterone/11-dehydrocorticosterone ratio and **H** the mineralocorticoid aldosterone and its precursor 11-deoxycorticosterone in the morning (7–8 am) and afternoon (6–7 pm) of GR^+/+^ and GR.^+/em4^ (*n* = 4 – 16) rats. The size measurements were evaluated using QuPath (vO.4.4), and the plasma concentrations were evaluated by two-way ANOVA and subsequently compared with an unpaired two-tailed *t* test with Welch’s correction. The values are presented as the mean ± SEMs. Differences were assessed at *P < 0.05 and **P < 0.01

There is increasing evidence that GR signaling is involved in salt-sensitive hypertension. Glucocorticoid excess in Cushing syndrome or glucocorticoid resistance in certain GR mutations induced hypertension and affected renal sodium retention [21]. Approximately 50% of patients with hypercorticolism and GR mutations exhibit hypertension (for review, see [55]). When kept on a high-salt diet, mice with reduced GR expression showed salt sensitivity and sustained hypertension that was attributed to increased mineralocorticoid receptor activation [24]. Similarly, rats with a mutation within the second zinc finger domain of the GR (Fig. 1A; GR^+/em2^ [52]) exhibited higher plasma corticosterone levels although salt-sensitive hypertension was only provoked in combination with high salt intake. These rats carried an out-of-frame mutation of the DNA-binding domain of the GR that resulted in an early Stop codon (GR^+/em2^) and thus represents a null allele (Figure S1A, B [52];). It has been furthermore reported that high salt intake activated the HPA axis, amplified the stress response, and altered glucocorticoid responsiveness in mice [6] indicating an interplay between salt intake, plasma cortisol/corticosterone, and tissue sensitivity to glucocorticoids.

Excess glucocorticoids stimulate renal sodium transport and can thus mediate mineralocorticoid-like effects although both receptors have distinct but also overlapping physiological functions (for review, see [55]). A recent study proposed that the GR is required for efficient aldosterone-induced transcription via the mineralocorticoid receptor [25, 59]. In line with these results, it has been demonstrated that mice with reduced global expression of GR (GR ^βgeo/+^) exhibit glucocorticoid resistance with increased plasma corticosterone levels and high-salt-induced hypertension, suggesting adaptive failure of the renal vasculature and tubules [24]. To decipher the role of renal GR in salt-sensitive hypertension, a nephron tubule-specific mouse GR knockout was generated that overall maintained the Na^+^ and K^+^ balance independent of the salt diet [4]. These mice did not present hypercorticosteronemia, and on a high-salt diet, their systolic blood pressure was not different from that of the controls, although these mutant mice showed a significant increase in diastolic blood pressure. This finding suggested that circulating plasma corticosteroids are involved in sodium homeostasis [4].

An orally active epoxide hydrolase inhibitor was provided to salt-loaded angiotensin-infused rats that exhibited salt-sensitive hypertension. This treatment decreased blood pressure and provided renal protection [23]. Indeed, epoxyeicosatrienoic acids (EETs) are important lipid mediators derived from arachidonic acid that can be further hydrolyzed to less active diols by the enzyme soluble epoxide hydrolase (sEH) [17]. There is increasing evidence that the inhibition of sEH increases the levels of EETs with anti-inflammatory and beneficial effects on metabolic diseases, including hypertension (for review, see [14, 54]). Although the mechanism by which these EETs prevent metabolic diseases is not completely understood, inflammation and chronic diseases, such as cardiovascular diseases, have been associated with increased sEH expression and accelerated conversion of omega-3 and omega-6 polyunsaturated fatty acids (PUFAs) [14]. Previously, GR haploinsufficient rats that developed salt-sensitive hypertension exhibited dysregulation of adrenal sEH, leading to a plasmatic elevation of several less active diol metabolites (DiHETrEs) highly suggested an association of sEH with primary generalized glucocorticoid resistance in rats with hypercortisolism and mineralocorticoid access [52].

To further elucidate the importance of the DNA-binding domain of the GR, we now studied a TALEN-engineered GR rat model lacking exon 3 (GR^+/em4^), which encodes the second zinc finger domain of the receptor [45] and is thus lacking the domains responsible for the nuclear localization, dimerization, and DNA binding but still maintains the ligand-binding domain (LBD) [42]. Following high salt exposure (6% for 5 weeks), these rats developed salt-sensitive hypertension accompanied by deregulated adrenal sEH, leading to a plasmatic increase in inactive omega-3 and omega-6 metabolites and altered aortic vascular reactivity. Our data demonstrated that mutations within the DNA-binding and dimerization domains of the GR predispose rats to salt-sensitive hypertension even in the absence of hypercortisolism and mineralocorticoid excess.

## Methods

### Animals

Male Sprague Dawley rats harboring the GR mutation (GR^+/em4^, EMMA stock number: EM:15187, [45]) were used throughout the study and generated by mating of heterozygous mutant male Sprague Dawley rats. To avoid possible interference with female hormones and cycles, experiments were only performed in males [9, 19]. Animal maintenance and all experimental procedures were approved by the Swiss Cantonal and Federal veterinarian authorities (license numbers VD3333a and VD 3776a) and were in agreement with the Swiss federal guidelines and ARRIVE recommendations [43]. All animals were housed in a humidity- (< 40%) and temperature-controlled room (23 ± 1 °C) with an automatic 12-h light/dark cycle (light: 7 am to 7 pm) in ventilated cages with free access to food (normal salt diet, NSD; 0.25% Na^+^ and 0.70% K^+^ diet; Provimi Kliba AG; number 3242-XP18, Switzerland, hereinafter referred to as the standard diet) and tap water in an approved animal care facility of the University of Lausanne. Genotyping was performed on ear biopsies at the age of 21 days using a DNA-based PCR strategy. The following primers were used: GR1a (intron 2-forward-3′-CTCTCAACATGGTAATTCATGTA-5′) and GR2b (intron 3-Reverse-3′-GCTGCTCAGACTCAGGCAC-5′) (Figure S1A). DNA biopsies were digested with proteinase K (Merck) overnight at 56 °C, and the DNA was extracted with (6 M) NaCl solution and precipitated with isopropanol/ethanol. The DNA was stored at – 20 °C until analysis. PCR was carried out using GoTaq DNA polymerase (Promega Corporation, Madison, WI) and primers (GR1a and GR2b). The PCR program was run for 39 cycles; each cycle consisted of 2.5 min at 94 °C, followed by 30 s at 59.9 °C and 6 min at 72 °C. PCR was used to generate a 962-base pair fragment for the wild type (GR^+/+^) and two fragments for the GR^+/em4^ rats: 962 and 653 bp. PCR products were separated and analyzed on a 2% agarose gel.

### Metabolic studies

Metabolic studies were performed as previously described [52]. Briefly, 5-week-old male rats were randomly divided into two different groups following (i) a standard NaCl diet (NSD, 0.25% Na^+^ w/w) or (ii) a high-salt diet comprising 6% NaCl (composed of 0.39% Na^+^ in water and 2.2% Na^+^ in food w/w) for 5 weeks (10-week-old group). The rats were placed in individual metabolic cages (Tecniplast, Buguggiate, Italy) for 4 to 5 days, and 1 day of adaptation was allowed at the beginning of the experiment. Physiological parameters such as body weight, food and water consumption, the amount of urine and feces, and the plasma and urinary electrolytes Na^+^ and K^+^ were measured at weeks 1 (days 1–5), 3 (days 16–21), and 5 (days 32–37). In between and during 10 consecutive days, the rats were placed in stock cages of two individuals. During metabolic cage experiments, the rats were examined daily for their general health and behavior. During the 10 days between collection, the rats were examined every 2 days. Throughout the experiment, several exclusion criteria were applied, e.g., more than 10% weight loss, shallow breathing, and anormal posture and activity. Notably, two out of the 24 rats were excluded from the experiments after the adaptation.

### Blood pressure measurements, blood sampling, and steroid analyses

Intraarterial blood pressure and heart rate were recorded between 2 and 4 pm as previously described [52]. Briefly, rats were anesthetized with a 1–2% isoflurane/oxygen mixture. The right carotid artery was exposed through a cervical incision and isolated. A catheter filled with a solution of glucose (5%) and heparin (3000 IU/ml) was inserted into the vessel. Following ligature around the artery, the catheter was subcutaneously tunneled to exit at the back of the neck. Following skin closure, rats were allowed to recover for 3 h. The arterial line was connected to a pressure transducer, and blood pressure and heart rate were recorded every 20 s for 15–30 min with a computerized data-acquisition system (Notocord HEM 3.1 software, SA, Croissy, France) at a sampling rate of 500 Hz [13, 60].

Blood samples were collected through cardiac or caudal vein puncture at 7–8 am or 6–7 pm into EDTA-coated tubes that were immediately placed on ice, centrifuged at 5000–6000 rpm for 20 min at 4 °C, and stored at – 20 °C. Omega-3 and omega-6 fatty acids were quantified by ultra-performance liquid chromatography-tandem mass spectrometry (UPLC‒MS) as previously described [52]. Steroids were quantified in positive or negative ionization mode using a 1290 UHPLC system coupled to a 6495 triple quadrupole instrument (Agilent) [50].

### GR-EGFR cross-talk assay

Primary rat embryonic fibroblasts from control (+ / + , wild-type), GR^em2/em2^ (− / − , knockout [52]) and GR^em4/em4^ (mutant) grown as described [46] in DMEM (Gibco BRL) supplemented with 5% heat-inactivated fetal bovine serum. For the GR(-activated) inhibition assay of the EGF-induced MAPK pathway, serum-starved cells (passages 3–4) were ± stimulated with EGF (10 ng ml^−1^, for 30 min) and/or DEX (10^−7^ M) as described previously [28].

### Cell culture experiments (COS-7 cells) and transfection assays

COS-7 cells (ATCC CRL-1651, Manassas, VA) were grown at 37 °C and 5% CO_2_ in DMEM supplemented with 10% heat-inactivated fetal bovine serum (FBS), penicillin G (100 IU/ml), streptomycin (100 μg/ml), and L-glutamine (2 mM). Wild-type rat GR (kind gift from David Pearce [31]) and GR^em4^ mutant constructs were generated according to standard molecular biology procedures (including introduction of silent restriction sites and em4 deletion, aa 470 to 508). COS-7 cells (∼ 5–8 × 10^4^ cells) were seeded on coverslips in a 24-well plate, and after 24 h (60–80% confluency), the cells were transfected with 0.15 μg of the wild-type GR or GR^em4^ construct according to the manufacturer’s instructions.

For the GR transactivation assays, COS-7 cells were plated in six-well plates at 2 × 10^5^ cells per well 2 days prior to transfection, grown to 80–90% confluence, and synchronized by daily medium change. One day before transfection, the cells were grown in medium containing charcoal-stripped serum. In total, 2 µg of pGL3 promoter reporter plasmid under the control of the PNMT-regulatory region was transfected using JetPrime (VWR) and the Dual-Luciferase Reporter Assay System (Promega) at a ratio of 4:1 according to the manufacturer’s instructions to normalize the transfection efficiency. After 24 h, the cells were harvested, and the extracts were prepared and assayed for firefly and Renilla luciferase activities via a dual luciferase reporter assay kit (Promega). The cDNAs encoding the rGR wild type, rGR^em2^, and rGR^em4^ were cloned and inserted into the p6R plasmid using standard molecular techniques; information about cloning is available upon request. The pPNMT-997/-466-LUC, pTAT3-TAT-LUC, and pGL-NF-kB-LUC constructs were used as previously described [1, 31].

### Treatment and immunofluorescence

Following transfection, the COS cells were incubated in medium supplemented with 10% charcoal heat-inactivated fetal bovine serum (FBS) for 24 h. After that, the cells were activated with 10 nM dexamethasone for 24 h. Activation was stopped by the addition of ice-cold PBS, after which the cells were immediately fixed with methanol for 20 min at – 20 °C and permeabilized for 1 min with acetone at – 20 °C. The cells were treated with blocking solution (20% fetal bovine serum, 5% bovine serum albumin, 0.05% gelatin) for 30 min at room temperature and incubated overnight at 4 °C with GR antibody (M-20; Santa Cruz Biotechnology, Inc., Santa Cruz, CA) diluted 1:150 in TBST (10 mM Tris, 150 mM NaCl, 0.1% Tween). The coverslips were washed three times in TBST buffer and incubated at room temperature for 1 h with fluorescein isothiocyanate-conjugated anti-rabbit antibody (Jackson ImmunoResearch, West Grove, PA) diluted 1:100. The coverslips were washed three times with TBST buffer and two times with PBS, dried, mounted on slides (SuperFrost Plus microscope slides; Fisher Scientific) in mounting solution, and examined with an upright fluorescence microscope.

### Western blot analyses

Cells, adrenal glands, and aortas were collected in RIPA buffer supplemented with protease and phosphatase inhibitors (Roche) and quantified by a BCA assay (Thermo Fisher). Briefly, 20–80 µg of protein was loaded and separated on a SDS‒PAGE gel (10% gradient gel for Western blot analysis for sEH and 7.5% gel for Western blot analyses for eNOS/NOS and PKG Bio-Rad Laboratories). The proteins were subsequently transferred to nitrocellulose membranes and probed with the following antibodies: mouse pERK (#91,065), rabbit ERK1/2 (#4695, Cell Signaling, dilution 1:1000), mouse α-tubulin (Sigma, T5168, dilution 1:4000), and mouse anti-Gapdh (MAB374, Merck, dilution 1:5000, anti-eNOS/NOS and anti-PKG, 1:1000); rabbit anti-sEH (10,010,146, Cayman Chemicals, dilution 1:500); mouse anti-eNOS/NOS (type III, 610,296, BD, dilution 1:200); and rabbit anti-PKG (ADI-KAP-PK005, Enzo Life Sciences, dilution 1:1000). Immunoreactive bands were visualized by chemiluminescence (ECL kit; Amersham Biosciences, 152 Little Chalfont, UK) or with an Odyssey infrared imaging system (LI-COR Biosciences, Germany). Fixation and visualization were performed on high-performance chemiluminescence film (Amersham Hyperfilm ECL, 28,906,839, Cytiva, Sweden) for the ECL method. Relative intensities were normalized through comparison to Gapdh content with ImageJ-8 bits.

### Soluble epoxide hydrolase activity assay

sEH activity was measured using the soluble epoxide hydrolase assay (ab240999; Abcam, Cambridge, UK) following the manufacturer’s instructions as described previously [52]. The results are presented in mUnits/mg.

### Histology

Paraffin sections of adrenal glands and thoracic aortas were stained with hematoxylin and eosin.

### Isolated vessel tension experiments

Ex vivo vasoreactivity was assessed using isolated vessel tension experiments. Aortas were cut into small rings of approximately 4 mm length and mounted on two 0.2-mm-diameter stirrups, passing through the lumen. Each vascular ring (two thoracic aorta rings per animal) was suspended in a vertical organ chamber filled with 10 ml of modified Krebs–Ringer bicarbonate solution, kept at 37 °C and aerated with 95% O_2_ and 5% CO_2_ at pH 7.4 as previously described [29]. Isometric tension was continuously recorded with a strain gauge system (PowerLab/8SP, AD Instruments, Oxford, UK). The vascular rings were progressively brought to their optimal resting tension by two cycles of 2.6 g stretching, equilibration, and washing. Potassium chloride (KCl, 100 mM) was then added to the organ chambers to test the viability of the vessels. After washing and equilibration, the vasoconstrictive properties of the vascular rings were assessed by the addition of cumulative doses of KCl (15–100 mM), angiotensin II (10^−10^ to 10^−6^ M), and phenylephrine (Phe, 10^−9^ to 10^−4^ M). Each dose‒response curve was followed by a washing and equilibration step. Indomethacin (10^−5^ M) was added to exclude possible interference from endogenous prostanoids, and Phe (10^−5^ M) was used to precontract the vessels. The endothelium-dependent vasorelaxation responses were assessed by the addition of cumulative doses of the endothelium-dependent vasodilator acetylcholine (ACh, 10^−9^ to 10^−4^ M). Finally, after washing, indomethacin and the eNOS inhibitor NG-nitro-L-arginine (10^−4^ M) were added to inhibit endogenous prostanoids and NO production. The vessels were again precontracted with Phe (10^−5^ M). Endothelium-independent relaxation was investigated using increasing doses of the nitric oxide (NO) donor 2-(N,N-diethylamino)-diazenolate-2-oxide diethylamine (DEA/NO 10^−10^ to 10^−4^ M). Unless otherwise stated, the solutions and drugs used were obtained from Sigma-Aldrich (Buchs, Switzerland). The resting tension (RT) was determined as the lowest tension achieved by each vascular ring during the entire experiment after the stretch/equilibration/wash steps. The residual tension (RDT) corresponded to the tension (after subtraction of the corresponding RT) following contraction with Phe (10^−5^ M), measured at the time the first dose of vasodilator was added. The vasoconstrictive response to KCl or Phe was expressed as gram of induced tension (after subtraction of the corresponding RT). The vasodilating response to ACh or DEA/NO was expressed as percent of the initial contraction induced by 10^−5^ M Phe (RDT). To assess and compare the global effect of each vasoactive agent on aortic rings from the different experimental groups, areas under the curve (AUCs) for KCl-, Phe-, ACh-, and DEA/NO-induced responses were calculated from the concentration‒response plots using GraphPad Prism version 9.0 (GraphPad Software, Inc., San Diego, CA, USA); the baseline was set at 0 and AUC was computed by Prism using the trapezoid rule. The half-maximal effective concentration (EC_50_) and maximum effect (*E*_max_) for each vasoactive agent were calculated from the dose–response curves obtained for each rat using GraphPad Prism 9.0. Five to seven animals were used per genotype and diet condition.

### Statistical analysis

All the data are presented as the mean ± SEM. G-power was applied prior to the experiments based on the data obtained in our previous study [36]. The data were evaluated by two-way ANOVA and compared with Tukey’s test, followed by comparison (condition per condition) with an unpaired two-tailed *t* test with Welch correction for all groups. In experiments with variable numbers, the data were evaluated by one-way ANOVA with Tukey’s multiple comparison test. Differences were assessed at *^,#,**†**^*P* < 0.05, **P < 0.01, ***P < 0.001, and ****P < 0.0001.

## Results

### Haploinsufficient GR^+/em4^ rats without hypercorticosteronemia presented with adrenal hyperplasia and impaired steroid synthesis

The mutant allele of the GR^+/em4^ rats lacked exon 3, which includes the second zinc finger domain of the GR, as evidenced by sequencing and genotyping (Fig. 1A, B; [45]). As shown in liver lysates, the 86–90 kDa truncated protein encoded by this mutant allele was expressed at the same level as the wild-type allele (Figure S1B). This resulted in a truncated DNA-binding domain of the GR, which, upon dexamethasone induction, was no longer able to delocalize from the cytoplasm to the nucleus, as shown in transfected COS cells (Figure S2A, B). Whereas DEX-induced EGF treatment of primary fibroblasts from control (+ / +) rats resulted in significant downregulation of the pERK/ERK ratio (normalized), EGFR signaling was not reduced in embryonic fibroblasts lacking GR (GR^em2/em2^) or expressing truncated GR (GR^em4/em4^) (Figure S2C, D). Furthermore, upon DEX induction in COS-7 cells, the GR^em4^ receptor failed to induce DNA-binding-dependent transcription of the TAT3- and PNMT-997/-466 promoters that contain GREs (Figure S2E, F), while fold repression did not differ between the GR^wt^ and GR^em4^-mutant receptors (Figure S2G). We therefore concluded that the mutant (GR^em4^) receptor failed to translocate but that transrepression was preserved.

We next monitored 5- to 6-week-old GR wild-type (control) and GR^em4/+^ rats for 5 weeks for physiological parameters such as weight, food and water intake, and urine and fecal excretion on a standard diet (NSD) or a high-salt diet (HSD) (Table S1). Under standard and high-salt diet conditions, both groups significantly gained weight over time. Water intake increased initially but decreased with time together with the food intake (Table S1). In both groups, HSD initially increased the urine volume, which decreased in the 5th week (Table S1). The fecal output was not different among the groups or among the conditions (Table S1). Under standard diet, Na^+^ and K^+^ excretion is decreased in GR^em4/+^ rats, albeit similar Na^+^ and K^+^ intake (Table S1). Overall, the physiological parameters did not differ in both groups.

On a standard diet, GR^+/em4^ rats presented adrenal hyperplasia with increased adrenal gland weight due to increased cortex and medulla size (Fig. 1C–F). Furthermore, on the same diet, the analyses of steroid hormones at the end of the active (7–8 am) and less active (6–7 pm) phase revealed a trend toward an increase in plasma corticosterone in the GR^+/+^ and GR^+/em4^ rats mainly at the end of the night phase (Fig. 1G, left panel). A similar pattern was observed for the inactive glucocorticoid 11-dehydrocorticosterone (Fig. 1G, middle panel), which is formed by 11β-hydroxysteroid dehydrogenase 2 (11β-HSD2) from the precursor of corticosterone. The ratio of corticosterone to 11-dehydrocorticosterone, a marker of 11β-HSD2 activity, was unchanged in both groups (Fig. 1G, right panel). The plasma concentrations of the mineralocorticoid aldosterone and its precursor 11-deoxycorticosterone did not differ between GR^+/+^ and GR^+/em4^ rats and only decreased in the wild-type rats in the afternoon (Fig. 1H, left and right panel, respectively). Plasma testosterone levels were decreased in the GR^+/em4^ rats, while androstenedione and progesterone did not differ between the groups (Table S2).

In summary, on NSD and compared with wild-type rats, GR^+/em4^ rats presented adrenal hyperplasia and steroid disturbances and showed a trend toward increased corticosterone levels. Overall, sodium and potassium homeostasis is maintained in both groups independent on the diet condition.

### GR^+/em4^ rats developed salt-sensitive hypertension accompanied by impaired sEH-hydrolized polyunsaturated omega-3 and omega-6 fatty acids synthesis

**Fig. 2:**
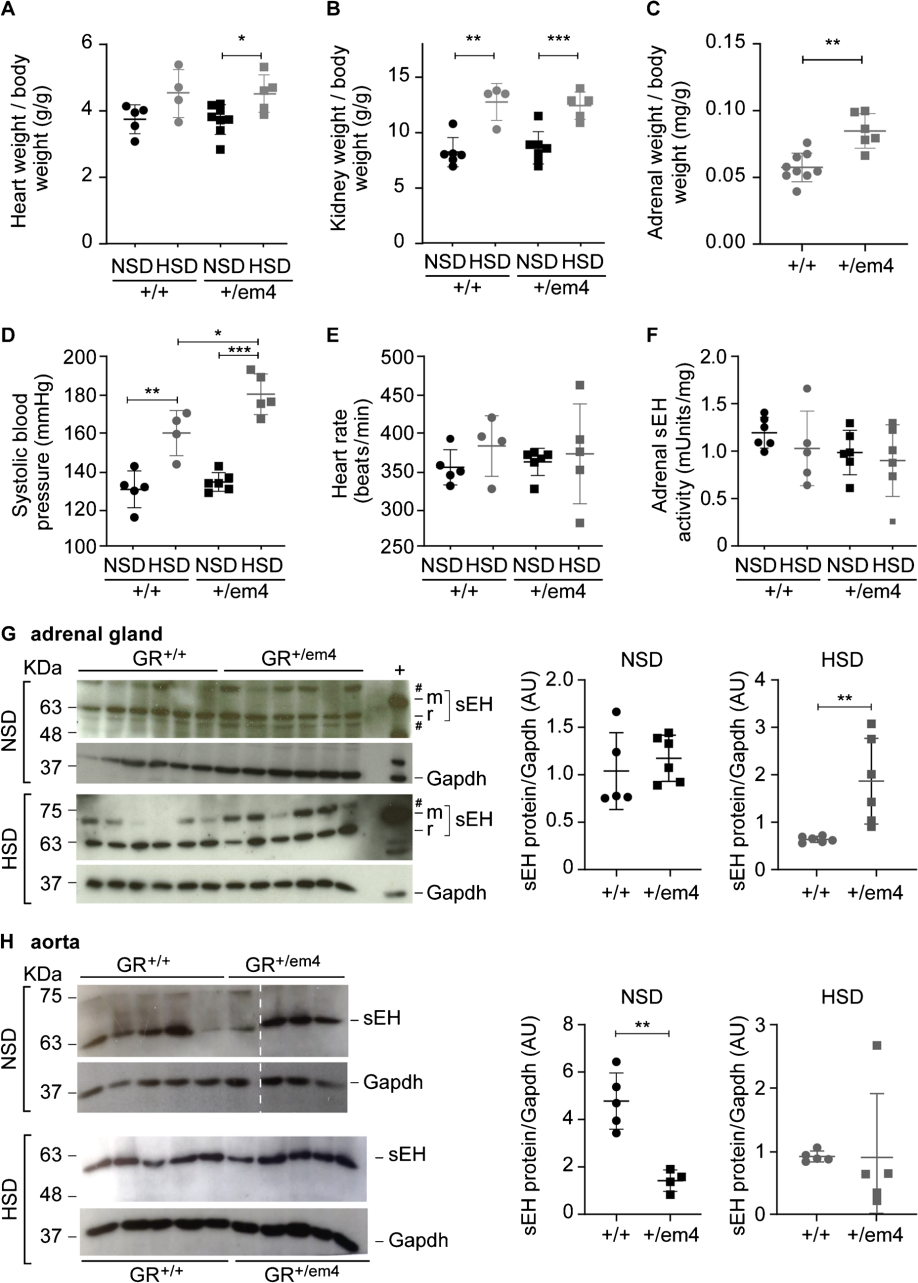
GR^+/em4^ rats developed salt-sensitive hypertension and altered adrenal and aortic sEH protein abundance.** A** Heart weight/body weight (g), **B** kidney weight/body weight on NSD and HSD (g), and **C** adrenal weight normalized to body weight (mg/g). **D** Systolic blood pressure and **E** heart rate on NSD and HSD (GR^+/+^, *n* = 4 or 5; GR^+/em4^, *n* = 5 or 6). **F** Measurement of adrenal sEH activity (mUnits/mg protein) under NSD (*n* = 6 per group and genotype) and HSD (GR^+/+^, *n* = 5; GR^+/em4^, *n* = 6) conditions. **G**,** H** Representative Western blot analyses of **G** adrenal (GR^+/+^, *n* = 6; GR^+/em4^, *n* = 6) and **H** aortic rat sEH protein abundance (r sEH, 64kD, GR^+/+^, *n* = 5; GR^+/em4^, *n* = 4) on a NSD (upper panel) or HSD (lower panel) and their quantification (left panels, NSD; right panels, HSD). The upper blot was cut (stripped line). The mouse sEH (m sEH; 64 kDa) was used as a positive control; ^#^unspecific bands. The values are presented as mean ± SEMs, and the data were evaluated via an unpaired two-tailed *t* test with Welch’s correction. Differences were assessed at *P < 0.05, **P < 0.01, and ***P < 0.001

**Fig. 3:**
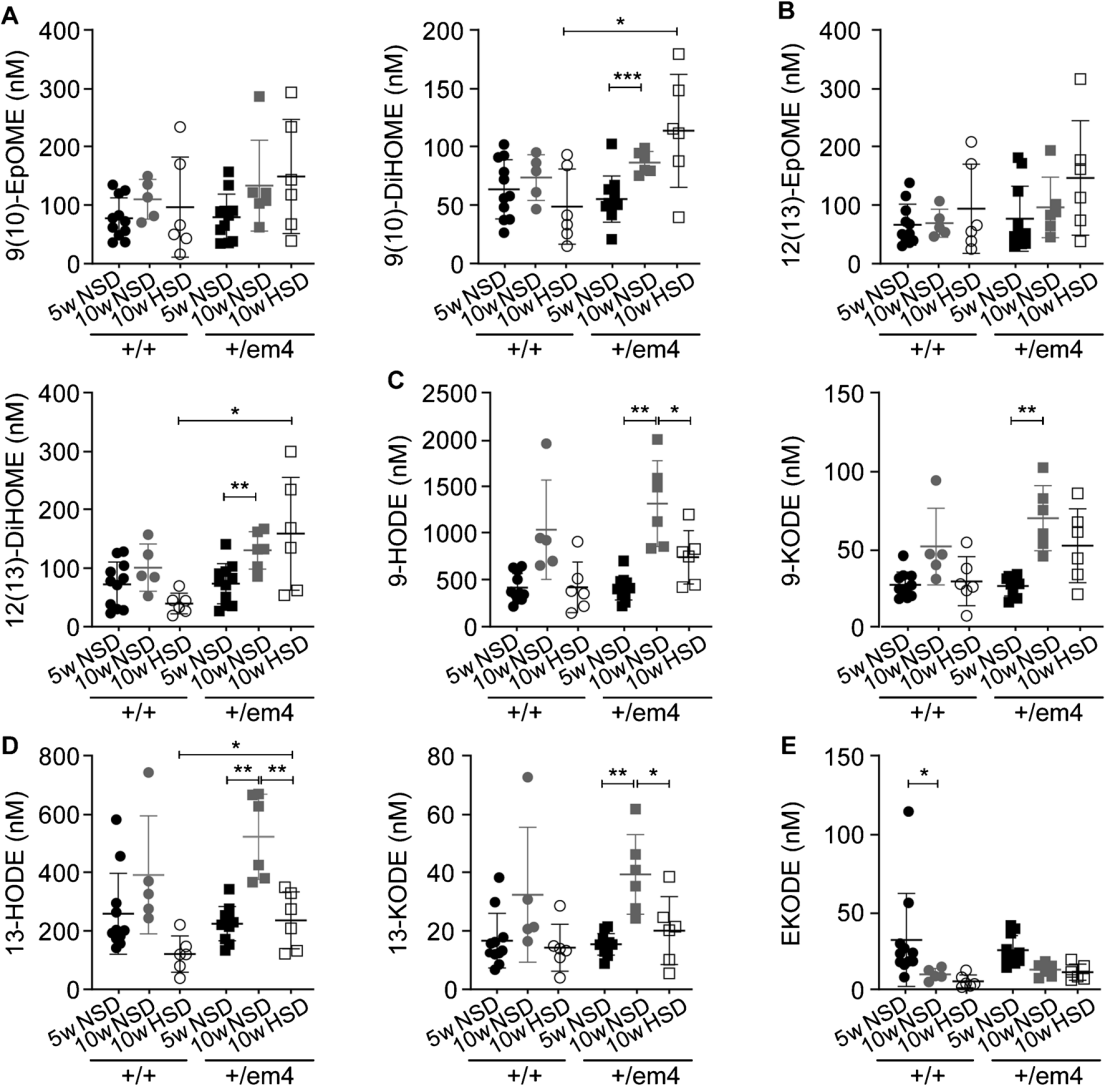
Increased inactive plasma metabolites of the linoleic, eicosapentaenoic, and docosahexaenoic acid pathways in GR^+/em4^ rats under high-salt diet.** A**–**E** Measurement of polyunsaturated fatty acid plasma metabolites of** A**,** B** the linoleic acid pathway and** C**,** D** the docosahexaenoic acid pathway in 5 (*n* = 12) and 10-week-old (*n* = 6; per diet and genotype) GR^+/+^ and GR.^+/em4^ rats fed standard (NSD) and high-salt (HSD) diets.** A**–**D** Active (epoxy-12Z-octadecenoic acid, 9(10)-EpOME, 12(13)-EpOME; hydroxoctadecadienoic acid, 9-HODE, 13-HODE; left) and less active (dihydroxy-9Z-octadecenoic acid, 9(19)-DiHOME; ketooctadecenoic acid, 9-KODE, 13-KODE; right panels) forms of derivatives and **E** epoxyketooctadecenoic acid (EKODE) are shown. The values are presented as the mean ± SEMs, and the data were evaluated via two-way ANOVA and subsequently compared with an unpaired two-tailed *t* test with Welch’s correction. Differences were assessed at *P < 0.05, **P < 0.01, and ***P < 0.001

On a standard diet, heart and kidney weight and the systolic blood pressure of the GR^+/em4^ rats were not different from that of the control rats (Fig. 2A, B, D). However, when challenged with a high-salt diet, both groups exhibited an increased adrenal and kidney weight (Fig. 2B, C), and GR^+/em4^ rats exhibited increased heart weight and higher systolic blood pressure compared to wild-type GR^+/+^ rats (Fig. 2A, D). The heart rate was not different among the groups and diet conditions (Fig. 2E). Independent of the diet, both groups showed similar epoxide hydroxylase activity per microgram protein in the adrenal gland (Fig. 2F). With respect to protein abundance, GR^+/em4^ rats did not differ from those fed a standard diet but did exhibit increased sEH protein abundance under high-salt conditions (Fig. 2G). We next analyzed the sEH-converted plasma metabolites of the linoleic, docosahexaenoic, and arachidonic acid pathways in 5- and 10-week-old GR wild-type and mutant rats under NSD conditions and found no differences. When challenged with a high-salt diet, the concentration of linoleic acids were significantly greater in 10-week-old GR^+/em4^ rats than in their control littermates (Table S3). An increase in the active form of linoleic hydroxyoctadecadienoic acid 9-HODE and in the inactive forms, namely, 9(10)-DiHOME, 12(13)-DiHOME, 9-KODE, and 13-KODE, was observed in the 10-week-old GR^+/em4^ rats (Fig. 3A–D). Furthermore, a high-salt diet increased the active form of 13-HODE and the inactive forms of 9(10)-DiHOME and 12(13)-DiHOME in GR^+/em4^ rats (Fig. 3A, B, and D). A decrease in epoxyketooctadecenoic acid (EKODE) was observed only in 10- versus 5-week-old GR wild-type rats (Fig. 3E).

**Fig. 4:**
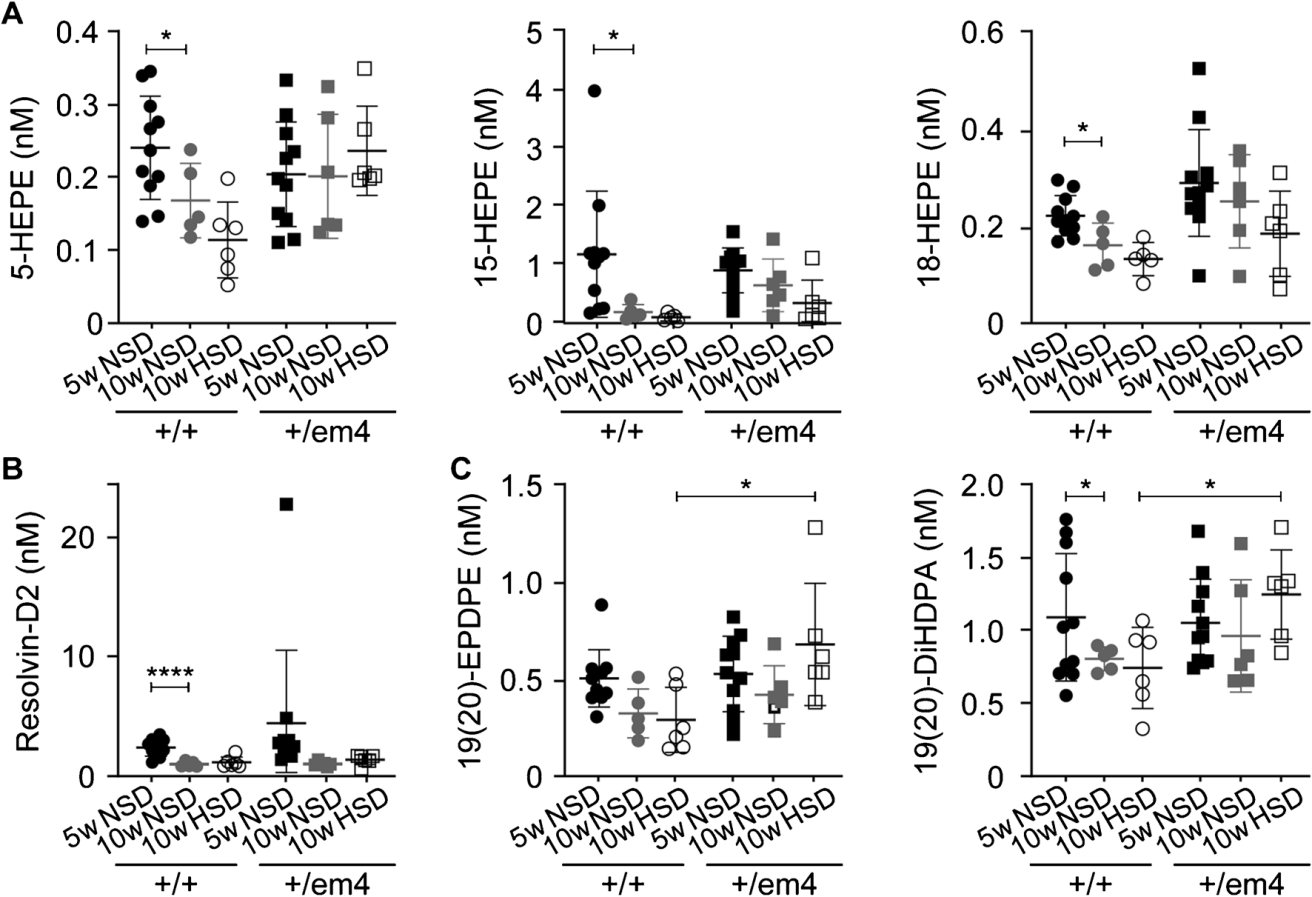
Increase in inactive plasma diol forms of the docosahexaenoic acid pathway in GR^+/em4^ rats. Measurement of polyunsaturated fatty acid metabolites from 5- (*n* = 6–11) and 10-week-old (*n* = 5–6, per group and genotype) GR^+/+^ and GR.^+/em4^ rats under standard (NSD) and high-salt (HSD) dies for **A** hydroxyeicosapentaenoic acid 5-HEPE, 15-HEPE, and 18-HEPE; **B** resolvin-D2; and** C** 19(20)-EPDPE and 19(20)-DiHDPA. Plasma metabolites were measured at the ages of 5 and 10 weeks under standard conditions and at 10 weeks under a high-salt diet. The values are presented as the mean ± SEMs, and the data were evaluated via two-way ANOVA and subsequently compared with an unpaired two-tailed *t* test with Welch’s correction. Differences were assessed at *P < 0.05 and ****P < 0.0001

**Fig. 5:**
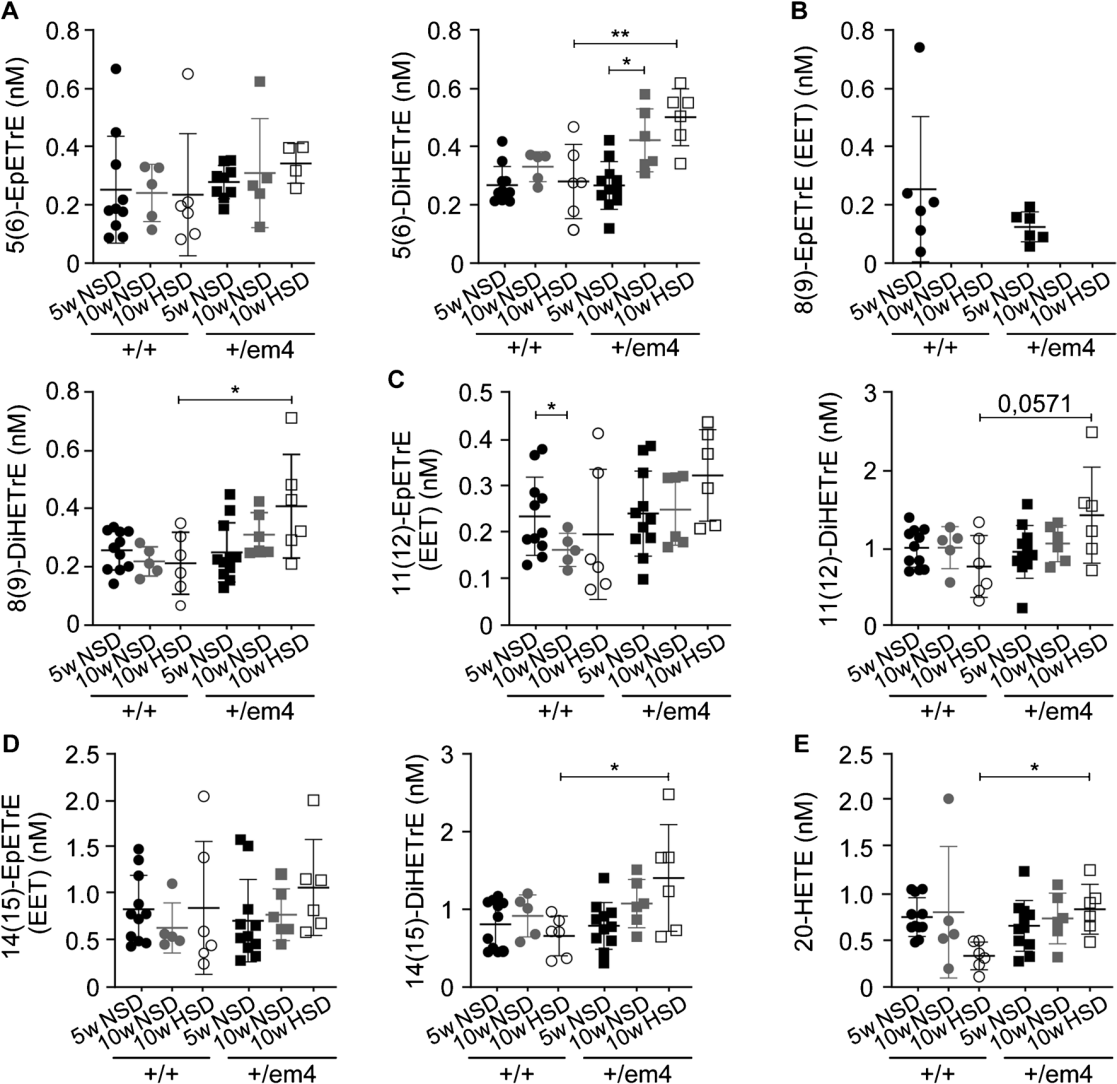
Increased levels of the less active plasma diol forms of EET and 20-HETE from the arachidonic acid pathway in GR^+/em4^ rats fed a high-salt diet. Measurement of polyunsaturated fatty acid metabolites from 5 (*n* = 6–11) and 10-week-old (*n* = 4–11, rats per group) GR^+/+^ and GR.^+/em4^ rats in the arachidonic acid pathway of **A** 5(6)-EpETrE (EET) and 5(6)-DiHETrE (nM); **B** 8(9)-EpETrE (EET) and 8(9)-DiHETrE (nM); **C** 11(12)-EpETrE (EET) and 11(12)-DiHETrE (nM**)**; **D** 14(15)-EpETrE (EET) and 14(15)-DiHETrE (nM) and **E** 20-HETE (nM) under standard (NSD) and high-salt (HSD) diet conditions. The values are presented as mean ± SEMs, and the data were evaluated via two-way ANOVA and subsequently compared with an unpaired two-tailed *t* test with Welch’s correction. Differences were assessed at *P < 0.05 and **P < 0.01

In wild-type rats fed a standard diet, metabolites of the docosahexaenoic acid pathway, such as hydroxyeicosapentaenoic acid 5-HEPE, 15-HEPE, 18-HEPE, resolvin-D2, and 19(20)-EPDPE, decreased with age (Fig. 4). In contrast, 10-week-old GR^+/em4^ rats exhibited increased levels of the plasma metabolites 19(20)-EPDPE and 19(20)-DiHDPA on HSD compared to wild type (Fig. 4C). An increase in the inactive diol form of 5(6)-DiHETrE was observed in GR^+/em4^ rats fed a standard diet (Fig. 5A). The epoxyeicosatrienoic acid 11(12)-EpETrE active metabolite was decreased in 10-week-old wild-type rats (Fig. 5C). In addition, the GR^+/em4^ rats fed a high-salt diet presented increased inactive plasma diol forms of 5(6)-DiHETrE, 8(9)-DiHETrE 14(15)-DiHETrE, and 20-HETE in the arachidonic acid pathway (Fig. 5A, B, D, E). The plasma metabolites of the eicosapentaenoic acid pathway did not differ between the genotypes and diet conditions (Table S3).

To summarize, GR^+/em4^ rats exhibited an increase in the inactive plasma diol forms of the linoleic, docohexaenoic, and arachidonic acid pathways, which is compatible with increased adrenal sEH protein abundance on a high-salt diet.

### On a standard diet, GR^+/em4^ rats showed increased thoracic aortic vascular response to phenylephrine and the NO donor DEA/NO, which was blunted under a high-salt diet

**Fig. 6:**
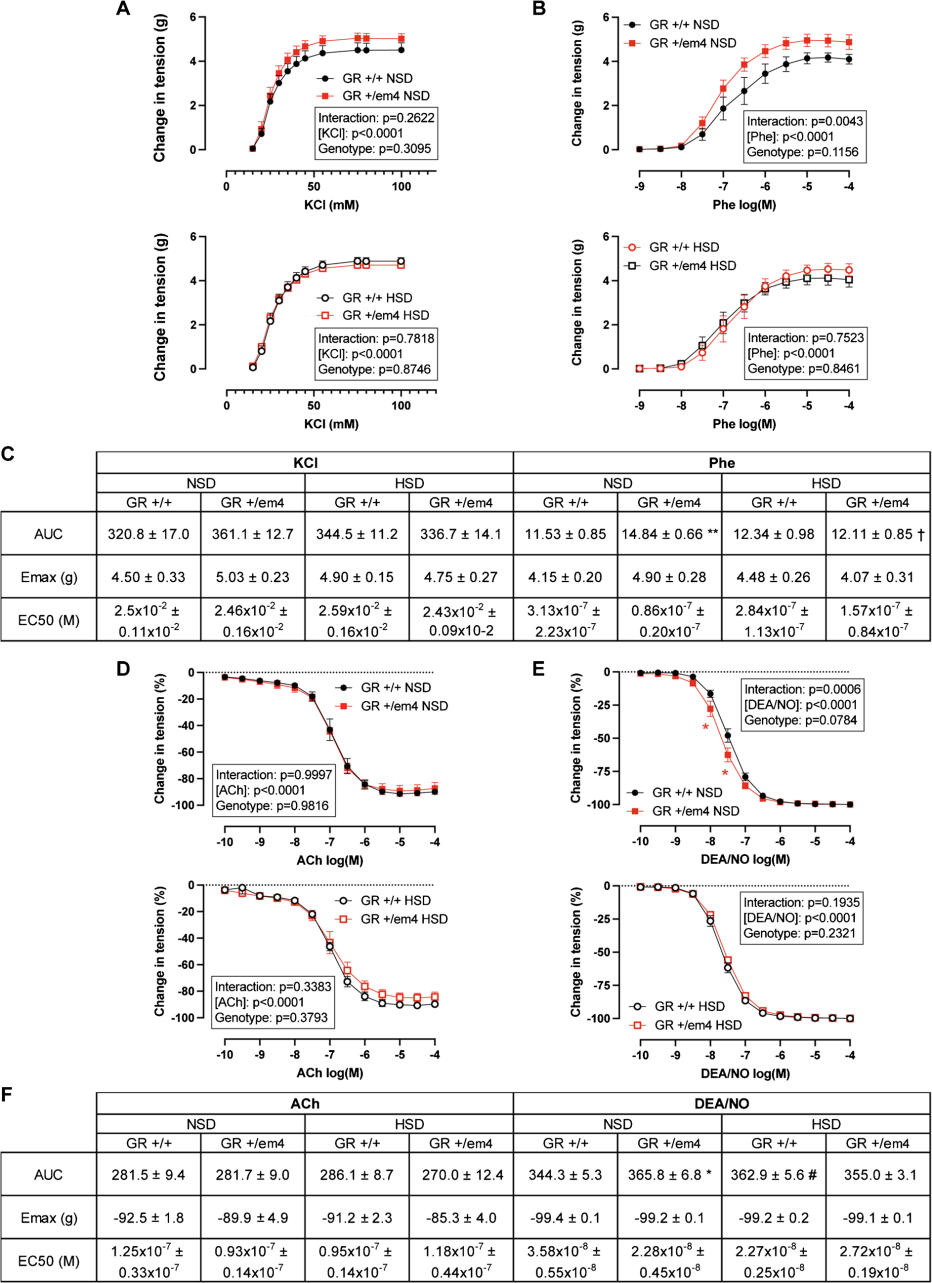
Altered aortic vasoreactivity in GR^+/em4^ rats under NSD but not HSD. **A**–**C** Pharmacological response of aortic rings to cumulative doses of **A** potassium chloride (KCl; NSD upper and HSD lower panel) and **B** phenylephrine (Phe; NSD upper and HSD lower panel). The vasoconstriction induced by KCl or Phe was expressed as gram of induced tension. **C** The values of the area under the curve (AUCs), maximum effects (*E*_max_), and the half-maximal effective concentrations (EC_50_) upon **A** KCl and **B** Phe stimulation. **D**–**F** Pharmacological responses of aortic rings to cumulative doses of **D** acetylcholine (ACh; NSD upper and HSD lower panel) or **E** the NO donor DEA/NO (NSD upper and HSD lower panel). The vasodilating response to ACh or DEA/NO was expressed as a percentage of the initial contraction induced by 10^−5^ M Phe. **F** The values of the area under the curve (AUCs), maximum effects (*E*_max_), and the half-maximal effective concentrations (EC_50_) upon **D** ACh and **E** DEA/NO stimulation. The data are presented as the mean ± SEM (*n* = 5–7 rats/group). The pharmacological curves were analyzed by two-way ANOVA with Tukey’s multiple comparison test using GraphPad Prism 9.0 (results of ANOVA are shown in the frame on each graph). The corresponding areas under the curve (AUCs), the half-maximal effective concentrations (EC_50_), and maximum effects (*E*_max_) are presented as means ± SEM. Student’s *t* test with Welch’s correction was used for direct comparisons between both genotypes or between the two diets. *Significant difference between the two genotypes under the same diet (NSD or HSD); † and # indicate significant differences between the HSD and NSD groups within the (†) GR^+/em4^ or (#) wild-type rats. The data were considered significant at *.^,#,†^*P* < 0.05 and **P < 0.01

Since polyunsaturated fatty acid (PUFA) metabolites are known to be involved in vasodilation and/or vasoconstriction, we first quantified the abundance of the sEH protein in thoracic aortas from 10-week-old wild-type and GR^+/em4^ rats fed a NSD or HSD (Fig. 2H, upper panel). On NSD, the abundance of the sEH protein was lower in GR^+/em4^ rats than in wild-type rats, whereas no difference was detected on HSD (Fig. 2H, lower panel). The vascular reactivity of the thoracic aortas was then investigated in organ chambers by testing their pharmacological response to potassium chloride (KCl), phenylephrine, acetylcholine, and the nitric oxide (NO) donor DEA/NO (Fig. 6). Resting tension was similar in all groups and under all diet conditions (Table 1). KCl induced a dose-dependent contraction of the aortic rings without any significant difference between the experimental groups (Fig. 6A). Phenylephrine induced a dose-dependent contraction in both genotypes, which under NSD was greater in GR^+/em4^ rats with larger AUC than in wild-type rats (Fig. 6B, C), while it was similar between both genotypes on HSD conditions. In the GR^+/em4^ rats, the response to phenylephrine was lower on HSD compared to NSD with a reduced AUC. Residual tension (RDT) was achieved in the aortic rings after precontraction with 10^−5^ M phenylephrine before the first dose of the vasodilator and did not significantly differ between genotypes and/or diets (Table 1). The dose–response to the endothelium-dependent relaxing agent acetylcholine was similar in all experimental groups (Fig. 6D). Under NSD conditions, the relaxation induced by DEA/NO was slightly but significantly increased in GR^+/em4^ rats compared to wild-type rats (Fig. 6E) with a greater AUC (Fig. 6F), while under HSD conditions, no difference was found between the two genotypes. Aortic rings from wild-type rats exhibited an increased response to DEA/NO under HSD conditions compared to NSD conditions with a greater AUC, while no difference between the two diets was found in mutant rats (Fig. 6E). Although statistically not significant, the *E*_max_ and EC_50_ values calculated for each vasoactive agent showed some trends and the *E*_max_ for phenylephrine tended to be higher in GR^+/em4^ rats under NSD than wild-type rats under NSD (*P* = 0.066) or than GR^+/em4^ rats under HSD (*P* = 0.079). Similarly, EC_50_ for DEA/NO tended to be lower in GR^+/em4^ rats than wild-type rats under NSD (*P* = 0.106) or in wild-type rats under HSD compared to NSD (*P* = 0.078). Endothelial nitric oxide synthase (eNOS) and cGMP-dependent protein kinase G (PKG) levels were similar in the thoracic aortas of wild-type and GR^+/em4^ rats, independent of the diet (Figure S3).

**Table 1:** Resting tension and precontraction of isolated aortic vascular rings

	NSD		HSD	
	GR^+/+^	GR^+/em4^	GR^+/+^	GR^+/em4^
Number of rats	5	5	7	6
RT	2.28 ± 0.03	2.33 ± 0.04	2.35 ± 0.03	2.30 ± 0.04
RDT before ACh	3.90 ± 0.19	4.51 ± 0.47	4.14 ± 0.34	3.64 ± 0.40
RDT before DEA/NO	5.61 ± 0.29	6.24 ± 0.28	5.88 ± 0.15	5.72 ± 0.32

*RT* resting tension, *RDT* residual tension achieved after precontraction with 10^−5^ M phenylephrine before adding cumulative concentrations of the vasodilator acetylcholine (ACh) (in the absence of the eNOS inhibitor NG-nitro-L-arginine, NLA) or DEA/NO (in the presence of NLA). The data are presented as the mean ± SEM. No statistically significant differences were found between genotypes or diet conditions according to two-way ANOVA with Sidak’s multiple comparison test

In summary, pharmacological stimulation of aortic rings with vasoconstrictors and vasodilators showed an increased vascular response of aortas from GR^+/em4^ rats to phenylephrine and DEA/NO under NSD conditions. This effect was blunted by the HSD.

## Discussion

### Rats with loss of the second zinc finger domain of the glucocorticoid receptor lacked increased plasma corticosteroid levels but presented with salt-sensitive hypertension

In the present study, we examined a new rat model (GR^+/em4^) where one GR allele lacked exon 3. This exon encodes for the second zinc finger, including the DNA-binding and dimerization domains [51, 53]. The functional albeit shorter protein has an abundance similar to that of the wild-type protein. The mutation resembled the human GR mutations R477H and R477S, which lie within the DNA-binding domain of the receptor and are associated with a lack of DNA binding and transactivation [48, 53, 56]. In humans, although decreased ligand binding, transactivation, and translocation are common features of the described GR mutations, no clear genotype‒phenotype relationship has been established (for review, see [50]). There might be a tendency that younger patients often present with severe clinical signs [57] although larger cohort studies are lacking. It will be interesting to use the rat as a model to study in the future the relationship of age and severity of the phenotype. On NSD, haploinsufficient GR^+/em4^ rats presented with normal systolic blood pressure, adrenal hyperplasia, and, on HSD, salt-sensitive hypertension and thus exhibited the phenotype of haploinsufficient GR^+/em2^ rats carrying one null allele [52]. Both GR rat models presented with normal blood pressure under a standard diet and changed to salt-sensitive hypertension upon high salt exposure, although the endocrine profiles were quite different. Whereas the GR^+/em2^ rats exhibited hypercorticosteronemia, which might be explained by the 50% decrease in GR protein abundance as a consequence of an impaired feedback control mechanism [36], the GR^+/em4^ rats did not exhibit significantly increased plasma corticosterone levels. The initial decrease in Na^+^ and K^+^ excretion in the GR^+/em4^ rats on a NSD might indicate an altered tubular GR activity that is not observed on HSD. To our knowledge, an animal model presenting such physiological features is unique. Additionally, in contrast to the haploinsufficient GR^+/em2^ rat model that presented increased aldosterone concentrations on NSD conditions (Fig. 1B; [52]), plasma aldosterone levels in GR^+/em4^ rats did not change. Interestingly, patients harboring GR mutations presented either with normal or high aldosterone levels [48, 55].

We propose that systemic rather than renal GR protein abundance is relevant for this negative feedback control [5, 24, 42], since nephron-tubule specific GR knockout mice did not exhibit hypercortironemia [4]. In humans, patients with various GR mutations and normal protein abundances presented with hypercortisolism, although the correlation between the kind of mutation, the GR protein abundance, and the cortisol level was not systematically documented [55]. Both GR^+/em2^ [52] and GR^+/em4^ rat models developed adrenal hyperplasia either only in the cortex (GR^+/em2^ [52]) or in the cortex and medulla (GR^+/em4^). In humans, approximately 40% of all GR mutations are linked to adrenal hyperplasia and documented at an age ranging from 3 to 71 years [55, 63]. Glucocorticoid resistance was linked to premature adrenarche in humans [40], characterized by an early expansion of the *zona fasciculata* and the formation of the *zona reticularis* [44]. More clinical data are needed to determine whether adrenal hyperplasia is a prerequisite for developing hypertension [55].

Overall, both GR rat models developed salt-sensitive hypertension, although they had different predispositions to standard diet, namely, hypercorticosteronemia in GR^+/em2^ [52] and normal plasma corticosteroid levels in GR^+/em4^ rats.

### The GR^+/em4^ rats differed in their adrenal soluble epoxide hydroxylase protein abundance and presented altered aortic vascular reactivity on a NSD that was blunted on an HSD

Upon HSD, both GR rat models presented an increase in the adrenal sEH protein abundance that was associated with an increase in hydrolyzed inactive forms of circulating omega-3 and omega-6 fatty acids [52] and which was exacerbated in the GR^+/em2^ rats. Indeed, soluble epoxide hydrolase (sEH) catalyzes the hydrolysis of polyunsaturated fatty acids to less active diols [27] and is implicated in metabolic and cardiovascular diseases [38]. However, in humans, the role of adrenal sEH is still largely unknown [11]. We found a large increase in oxidation products derived from linoleic acid (LA) and arachidonic acid (AA) and the docosahexaenoic acid (DHA), eicosapentaenoic acid (EPA), and alpha-linolenic acid (ALA) pathways. These bioactive lipids, also called oxylipins, are potent endogenous mediators involved in the regulation of various biological processes. Interestingly, on a standard diet, a decrease in several of these active metabolites was observed in wild-type rats with age, while remained unchanged in GR^+/em4^ rats. Oxylipin biosynthesis has been previously linked to aging and even cellular senescence [61], and transgenic expression of cyclooxygenase 2 (COX2) caused premature aging phenotypes in mice [26]. Furthermore, epoxyeicosatrienoic acids (EETs) are essential to maintain water and electrolyte homeostasis, and the inability to increase EETs in response to high-salt diet resulted in salt-sensitive hypertension [22]. In renal tubular epithelial cells, 5(6)-EET inhibited apical sodium transport, while 11(12)-EET induced a direct natriuresis [38], and both EETs were not upregulated in GR^+/em4^ rats following a HSD. On a HSD, 10 week-old GR^+/em4^ rats exhibited an increased number of the less active forms of fatty acids compared to wild-type rats. Recently, angiotensin II infusion of wild-type C57BL/6J mice increased the levels of proinflammatory oxylipins, including hydroxyeicosatetraenoic acids (HETEs) and dihydroxyoctadecenoic acids (DiHOMEs), which are implicated in immune responses and cytotoxic processes and thus contribute to senescence and oxidative stress [20]. Increased plasma linoleate diols were also found to be increased in patients with severe COVID-19, which was proposed as a pathological signature [33]. Indeed, impaired sEH-hydrolyzed metabolites might have contributed to the salt-sensitive hypertension observed in our present study. Upon HSD, increased levels of the major metabolite of the arachidonic acid pathway, 20-HETE, were observed in GR^+/em4^ rats but not in GR^+/em2^ rats [52]. This metabolite plays an important role in the regulation of vascular tone in arterioles of the kidney, brain, heart, and skeleton [35] and is a potent vasoconstrictor by sensitizing vascular smooth muscle cells to constrictor stimuli [39]. It might thus augment the sensitivity of mesenteric arteries to phenylephrine and induce contraction, as reported in spontaneously hypertensive Wistar-Kyoto rat [64]. In addition, several studies have demonstrated a role for 20-HETE in the early stage of salt-sensitive hypertension [15, 58]. The decreased response of aortas from GR^+/em4^ rats to phenylephrine following HSD exposure might be explained by a permanent contractile state and thus a loss of efficiency of phenylephrine. Although wild-type rats exhibited an increased response to DEA/NO under HSD compared to NSD conditions, no change was observed in GR^+/em4^ rats. This might indicate vascular dysfunction, as described in a model of androgen-dependent hypertension [62]. Interestingly, GR^+/em4^ rats exhibited increased heart weight following a HSD, which might indicate a hypertensive state and the result of volume overload as observed in humans [49]. In our study, the assessment of the aortic vasoreactivity revealed changes in the response of vascular rings to phenylephrine and DEA/NO, but not to KCl and acetylcholine, although *E*_max_ and EC_50_ were not significantly different between groups and diet conditions. This is likely due to the large variability within one group and the limited number of experimental animals. On the other side, a more subtle phenotype may be even closer to what is found in the human population harboring GR mutations. At this stage, we cannot determine whether the altered vascular reactivity in the mutant rats was a cause or consequence.

Patients harboring GR mutations present with a large spectrum of clinical symptoms, but which kinds of GR mutations are causative and whether high-salt diet exposure predisposes patients to develop hypertension is not known. Patients with hypertension exhibit low plasma levels of arachidonic acid (AA), eicosapentaenoic acid (EPA), and docosahexaenoic acid (DHA) and show an inverse association between plasma PUFA content and blood pressure [8, 18]. Hypertension remains highly prevalent (~ 50%) in patients with glucocorticoid resistance, although the underlying mechanism is not known**.** The study of GR-modified rat models revealed complex interactions between impaired GR signaling, action of epoxy hydrolases and their converted metabolites, and the vessels as target organ. Such models might further help to elucidate whether GR-mediated hypertension is primarily due to sodium and water retention, vascular dysfunction, or both. Mice in which the GR was lacking in the vascular endothelium were resistant to dexamethasone-induced hypertension [16]. Determination of the baseline levels of plasma PUFAs in hypertensive patients might serve as a diagnostic marker for a potential predisposition to developing salt-sensitive hypertension.

## Supplementary Information

Below is the link to the electronic supplementary material.

Supplementary file1 (DOCX 910 KB)

## Data Availability

Data is provided within the manuscript or supplementary information files. Besides, supplementary information/data can be sent by authors if needed by reviewers or readers. However, uncropped blots have been sent to reviewers.

## Abbreviations

ACTH: Adrenocorticotropic hormone
DiHETrE: Dihydroxyeicosatrienoic acid
DiHOME: Dihydroxy-9Z-octadecenoic acid
EET: Epoxyeicosatrienoic acid
EpETrE: Epoxyeicosatrienoic acid
GR: Glucocorticoid receptor
HETE: Hydroxyeicosatetraenoic acid
KODE: Ketooctadecenoic acid
sEH: Soluble epoxide hydrolase
