# Supplementary material for: Salt-sensitive hypertension in GR mutant rats is associated with altered plasma polyunsaturated fatty acid levels and aortic vascular reactivity
Source: Pflugers Arch. 2024 Sep 10;477(1):37–53. doi: 10.1007/s00424-024-03014-y (PMC11711871; doi:10.1007/s00424-024-03014-y)
Supplement: Supplementary file 1 — Supplementary file1 (DOCX 910 KB) [file 424_2024_3014_MOESM1_ESM.docx]

**Supplementary file**

**Salt-sensitive Hypertension in GR Mutant Rats is associated with Altered Plasma Polyunsaturated Fatty Acid levels and Aortic Vascular Reactivity**

**Pflügers Archiv – European Journal of Physiology**

Verouti S.^1,2,3^, Aeschlimann G.^1^, Wang Q.^4^, Ancin Del Olmo, D.^1^, Peyter AC^5^, Menétrey S^5^, Winter DV.^6^, Odermatt A.^6,^, Pearce, D.^7^, Hummler E.^1,2^ and Vanderriele PE.^1,2*^

^1^ Department of Biomedical Sciences, University of Lausanne, Lausanne, Switzerland

^2^ National Center of Competence in Research, Kidney.CH, Lausanne, Switzerland

^3^ Department for BioMedical Research (DBMR), University of Bern, Bern, Switzerland

^4^ Division of Nephrology and Hypertension, Lausanne University Hospital (CHUV), Lausanne, Switzerland

^5^ Neonatal Research Laboratory, Clinic of Neonatology, Department Woman-Mother-Child, CHUV, Lausanne, Switzerland

^6^ Division of Molecular and Systems Toxicology, Department of Pharmaceutical Sciences, University of Basel, Basel, Switzerland

^7^ Department of Medicine and Cellular & Molecular Pharmacology, University of California, San Francisco, USA

***Correspondence to** Dr. Paul-Emmanuel Vanderriele, Department of Biomedical Sciences, University of

Lausanne, Lausanne, Switzerland; Email: paul-emmanuel.vanderriele@unil.ch.

**Supplemental Table 1:**

**Physiological parameters of GR** **wildtype and GR^+/em4^ rats following standard diet (NSD) and high salt diet (HSD)**

|  |  | GR^+/+^ | | GR^+/em4^ | |
| --- | --- | --- | --- | --- | --- |
| Parameters | Exp. Period | GR^+/+^ NSD | GR^+/+^ HSD | GR^+/em4^ NSD | GR^+/em4^ HSD |
| Weight (g) | Day 1-5 | 214 ± 2 | 222.4 ± 3 | 232.47 ± 36,09^§^ | 225.99 ± 35.56 |
|  | Day 16-21 | 359.2 ± 23.8^┼┼┼^ | 340.3 ± 33.8^┼┼┼^ | 370.08 ± 42.56^┼┼┼^ | 334.23 ± 44.21^┼┼┼^ |
|  | Day 32-37 | 423.88 ± 29.87^***^ | 403.86 ± 28.84^***^ | 437.83 ± 43.70^***^ | 419.09 ± 28.76^***^ |
| Food intake (g)/body weight (g) ratio | Day 1-5 | 0.10 ± 0,01 | 0.07 ± 0,02^ǂǂǂ^ | 0.09 ± 0.01^§^ | 0.072 ± 0.01^ǂǂǂ^ |
|  | Day 16-21 | 0.07 ± 0,01 | 0.08 ± 0,01^ǂǂǂ^ | 0.07 ± 0.01 | 0.08 ± 0.01^ǂǂ^ |
|  | Day 32-37 | 0.05 ± 0,01 | 0.05 ± 0,02 | 0.05 ± 0.01 | 0.07 ± 0.01^ǂǂǂ^ |
| Water intake (g)/ body weight (g) ratio | Day 1-5 | 0.12 ± 0,02 | 0.35 ± 0.14^ǂǂǂ^ | 0.12 ± 0.03^§^ | 0.41 ± 0.10^ǂǂǂ^ |
|  | Day 16-21 | 0.10 ± 0,06^┼^ | 0.35 ± 0.08^ǂǂǂ^ | 0.10 ± 0.05 | 0.37 ± 0.09^ǂǂǂ^ |
|  | Day 32-37 | 0.07 ± 0,03 | 0.18 ± 0.09^***, ǂǂǂ^ | 0.06± 0.03^*^ | 0.23 ± 0.07^***, ǂǂǂ^ |
| Urine excretion (g)/body weight (g) ratio | Day 1-5 | 0.04 ± 0,02 | 0.26 ± 0.1^ǂǂǂ^ | 0.04 ± 0.02 | 0.27 ± 0.07^ǂǂǂ^ |
|  | Day 16-21 | 0.03 ± 0,01 | 0.24 ± 0.07^ǂǂǂ^ | 0.04 ± 0.01 | 0.27 ± 0.08^ǂǂǂ^ |
|  | Day 32-37 | 0.04 ± 0,02 | 0.15 ± 0.06^***, ǂǂǂ^ | 0.03 ± 0.01 | 0.21 ± 0.09^**, ǂǂǂ^ |
| Feces (g)/body weight (g) ratio | Day 1-5 | 0.04 ± 0,01 | 0.03 ± 0.01 | 0.04 ± 0.01 | 0.029 ± 0.01 |
|  | Day 16-21 | 0.03 ± 0,00 | 0.03 ± 0.01 | 0.03 ± 0.00 | 0.032 ± 0.01 |
|  | Day 32-37 | 0.03 ± 0,00 | 0.03 ± 0.01 | 0.02 ± 0.01 | 0.028 ± 0.01 |
| 24h Na^+^ intake (mmol L/24h/g of body weight) | Day 1-5 | 0.01 ± 0.0006 | 0.09 ± 0.04 | 0.01 ± 0.001 | 0,11 ± 0.04 |
|  | Day 32-37 | 0.007 ± 0.02°°° | 0.07 ± 0.03°° | 0.005 ± 0.002° | 0.07 ± 0.03°°° |
| 24h Na^+^ excretion (mmol L/24h/g of body weight) | Day 1-5 | 0.01 ± 0.002 | 0.02 ± 0.01 | 0.005 ± 0.001^§^ | 0.02 ± 0.004 |
|  | Day 32-37 | 0.004 ± 0.001° | 0.02 ± 0.01° | 0.005 ± 0.002 | 0.02 ± 0.004°° |
| K^+^ excretion (mmol L/ 24h/g of body weight) | Day 1-5 | 0.02 ± 0.004 | 0.002 ± 0.001 | 0.01 ±0.001^§^ | 0.002 ± 0.001 |
|  | Day 32-37 | 0.01 ± 0.001° | 0.003 ± 0.0027 | 0.01 ±0.004 | 0.003 ± 0.002 |
| Na^+^ in plasma (mM) | Day 1-5 | 132.36 ± 1.59 | 130.18 ± 18.11 | 132 ± 2.99 | 137.95 ± 3.33^ǂ^ |
|  | Day 32-37 | 136.16 ± 1.88 | 141.16 ± 3^ǂǂ^ | 133.46 ± 2.24 | 140.1 ± 2.54^ǂǂǂ^ |
| K^+^ in plasma (mM) | Day 1-5 | 5.77 ± 0.77 | 5.45 ± 1.86 | 5.77 ± 0.58 | 5.95 ± 0.71 |
|  | Day 32-37 | 6.10 ± 1.39 | 5.66 ± 2.24 | 6.63 ± 0.26 | 5.3 ± 0.42 |

Statistical significance was measured using *t*-test with Welch’s correction for group to group comparison. Data are shown as mean ± SEM. 6 rats were used per genotype and diet; NSD, standard diet (0.25 % Na^+^, 0.7 % K^+^), HSD, high salt diet (2.2 % Na^+^, 0.7 % K^+^); *^,┼,°^ indicate significant differences between the experimental period of day 32-37 and day 16-21 (*) and, day 16-21 and day 1-5 (^┼^) and, day 1-5 and day 32-37 (°). ǂ indicates significant differences between high salt diet (HSD) and standard diet (NSD); § indicates significant differences between the genotypes. Data were considered significant at *,┼,ǂ,§,°, *P* <0.05; ǂǂ,**,°°, *P* <0.01; ***^,┼┼┼^,ǂǂǂ ,°°°, *P* <0.001.

**Supplemental Table 2.**

**List of plasma steroid hormones tested in wildtype and GR^em4/+^ rats following standard (NSD) diet condition**

| Hormones | Time | n | GR^+/+^ | n | GR^+/em4^ |  |
| --- | --- | --- | --- | --- | --- | --- |
| Testosterone  (nM) | am | 8 | 0.81 ± 0.50 | 8 | 0.88 ± 0.53 |  |
|  | pm | 4 | 0.88 ± 0.37 | 4 | 0.41 ± 0.11* |  |
| Androstenedione  (nM) | am | 8 | 0.53 ± 0.27 | 8 | 0.49 ± 0.19 |  |
|  | pm | 4 | 0.69 ± 0.06 | 4 | 0.55 ± 0.10 |  |
| Progesterone  (nM) | am | 8 | 6.50 ± 3.35 | 8 | 3.96 ± 2.70 |  |
|  | pm | 4 | 4.41 ± 1.54 | 4 | 7.76 ± 5.36 |  |

Statistical significance was measured using GraphPad Prism version 9.0 (GraphPad Software, Inc, San Diego, CA, USA) with *t*-test with Welch’s correction for comparison of two groups. Data are shown as mean ± SEM; n indicates the number of animals (4-8). Blood was recovered at 7:00 – 8:00 (am) and 6:00 – 7:00 (pm); Data were considered significant at **P* <0.05 (am vs pm).

**Supplemental Table 3.**

**Concentrations of α- and γ-linoleic acids and metabolites of the eicosapentanoic acid pathway in 5-week- and 10-week-old wildtype and GR^+/em4^ rats following standard (NSD) and high salt diet (HSD)**

|  |  | GR^+/+^ | | | GR^+/em4^ | | |
| --- | --- | --- | --- | --- | --- | --- | --- |
| Pathway | Metabolites | 5 w NSD | 10 w NSD | 10 w HSD | 5 w NSD | 10 w NSD | 10 w HSD |
| Linoleic acid | α- linoleic acid (nM) | 1111.30 ± 143.47 | 1065.06 ± 137.24 | 884.26 ± 251.71 | 1142.02 ± 206.05 | 1264.90 ± 192.43 | 1372.24 ± 223.46^**^ |
|  | γ- linoleic acid (nM) | 114.03 ± 39.23 | 84.68 ± 38.32 | 72.62 ± 47.11 | 106.81 ± 36.49 | 108.19 ± 45.56 | 170.66 ± 75.58 |
| Eicosapentanoic acid pathway | 14(15)-DiHETE (nM) | 0.05 ± 0.03 | 0.04 ± 0.01 | 0.04 ± 0.01 | 0.07 ± 0.04 | 0.04 ± 0.01 | 0.05 ± 0.02 |
|  | 17(18)-DiHETE (nM) | 0.32 ± 0.13 | 0.28 ± 0.04 | 0.28 ± 0.12 | 0.37 ± 0.15 | 0.34 ± 0.10 | 0.37 ± 0.06 |

Statistical significance was measured using GraphPad Prism version 8.059.0 (GraphPad Software, Inc, San Diego, CA, USA) and 2-Ways ANOVA for continuous data and *t*-test with Welch’s correction for comparison group per group. Data are shown as mean ± SEM; n = 6 animals were used per diet, condition and genotype; NSD, standard diet (0.25 % Na^+^, 0.7 % K^+^) during 5 weeks (5w) and 10 weeks (10w), HSD, high salt diet (2.2 % Na^+^, 0.7 % K). DiHETE (dihydroxyicosatetraenoic acid); ^*^ indicates significant differences between the genotypes on HSD. Data were considered significant at *P* <0.05; ^**^ *P* <0.01.

**Supplemental Figure 1**


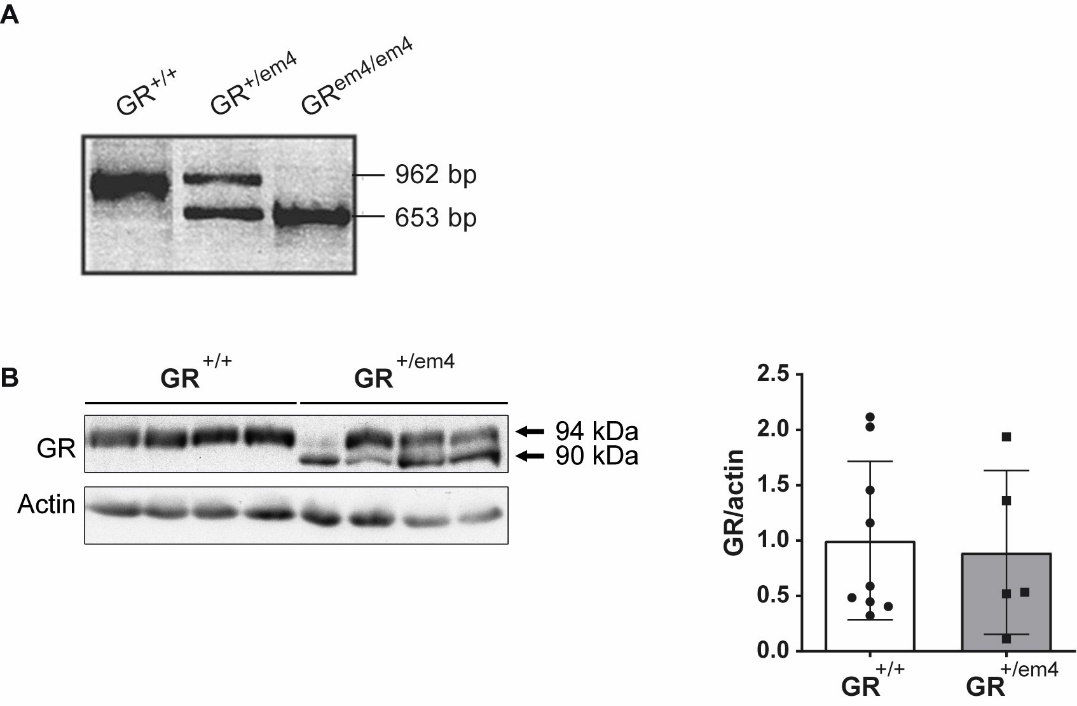


**The mutated glucocorticoid receptor (GRem4) showed normal protein abundance. (A)** Genotyping of wildtype GR (+/+), heterozygous mutant GR (+/em4) and homozygous mutant GR (em4/em4) embryos indicating the wildtype (962bp) and mutant (653bp) GR alleles. PCR was run on an agarose gel (2%). (**B**) Representative Western blot analysis of liver lysates from wildtype GR (+/+) and heterozygous mutant GR (+/em4) rats; actin was used as a loading control and quantification. Results are presented as mean ± SEM. Data were analyzed using One-way ANOVA with Tukeys’ multiple comparison test, (GR^+/+^, n = 9; GR^+/em4^, n = 5 rats).

**Supplemental Figure 2**


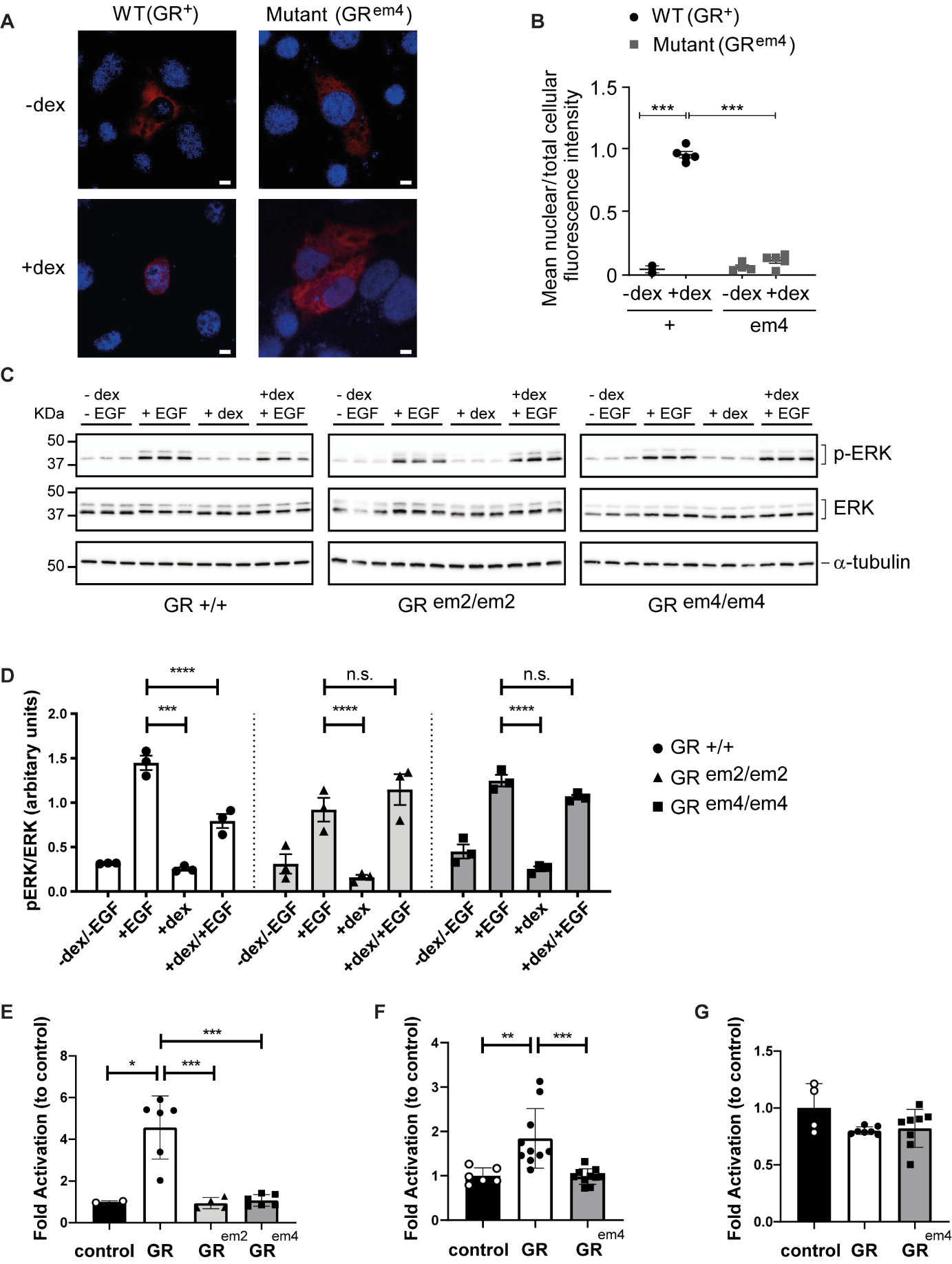


**Localization of fluorescent-labeled GR^em4^ in COS cells.** (A) Presence of immunofluorescent-labelled transfected wildtype (red; GR^+^, control, left) and mutant (GR^em4^, right panels) construct in COS cells ± treated with dexamethasone (10^-7^ M, 1 h). Scale bar, 15 µm. (**B**) Quantification of the mean nuclear per cellular fluorescence intensity in COS cells transfected with wildtype (GR) and GR^em4^ construct. Data are indicated as mean ± SEM, evaluated by two-way ANOVA and compared with unpaired two-tailed *t* test with Welch’s correction. Differences were assessed at *** *P* < 0.001. (**C**) Representative Western blot analyses of rat fibroblasts (n = 3; triplicates) (wildtype (+/+), knockout (-/-; GR^em2/em2^ **[36]** and homozygous mutant GR^em4/em4^) treated with ± EGF (10 ng ml^-1^), ± DEX (100 nM) or both (for 30 min). Protein extracts were immunoblotted for ERK, pERK and α-tubulin. (**D**) Quantification of Western blot data indicating the pERK/ERK protein ratio. Data are indicated as mean ± SEM, evaluated by one-way ANOVA with Tukeys’ multiple comparison test; n.s., not significant; ***,*P* <0.001; ****,*P* < 0.0001. **(E,F)** Fold transactivation and **(G)** transrepression activity of (**E**) GR^wt^ (GR), GR^em2^ (ko) and GR^em4^ and, (**F,G**) GR^wt^ (GR) and GR^em4^ receptors using the 5’-regulatory region of **(E)** rat pTAT3-TAT-LUC - (n = 2-6), **(F)** pPNMT-997/-466-LUC- (n = 6-12) and, **(G)** pGL-NF-kB-LUC (n = 4-8) reporter constructs in the presence of **(E,F)** 10^-7^ M dex and, **(G)** 10^-7^ M dex and 10 ng/ml TNFα stimulation; values are expressed as fold activation over empty vector upon dex stimulation. Significances were analyzed by One-way ANOVA with Tukey’s multiple comparison test. **,P* < 0.05; ***,P* < 0.01; ****,P*< 0.001.

**Supplemental Figure 3**


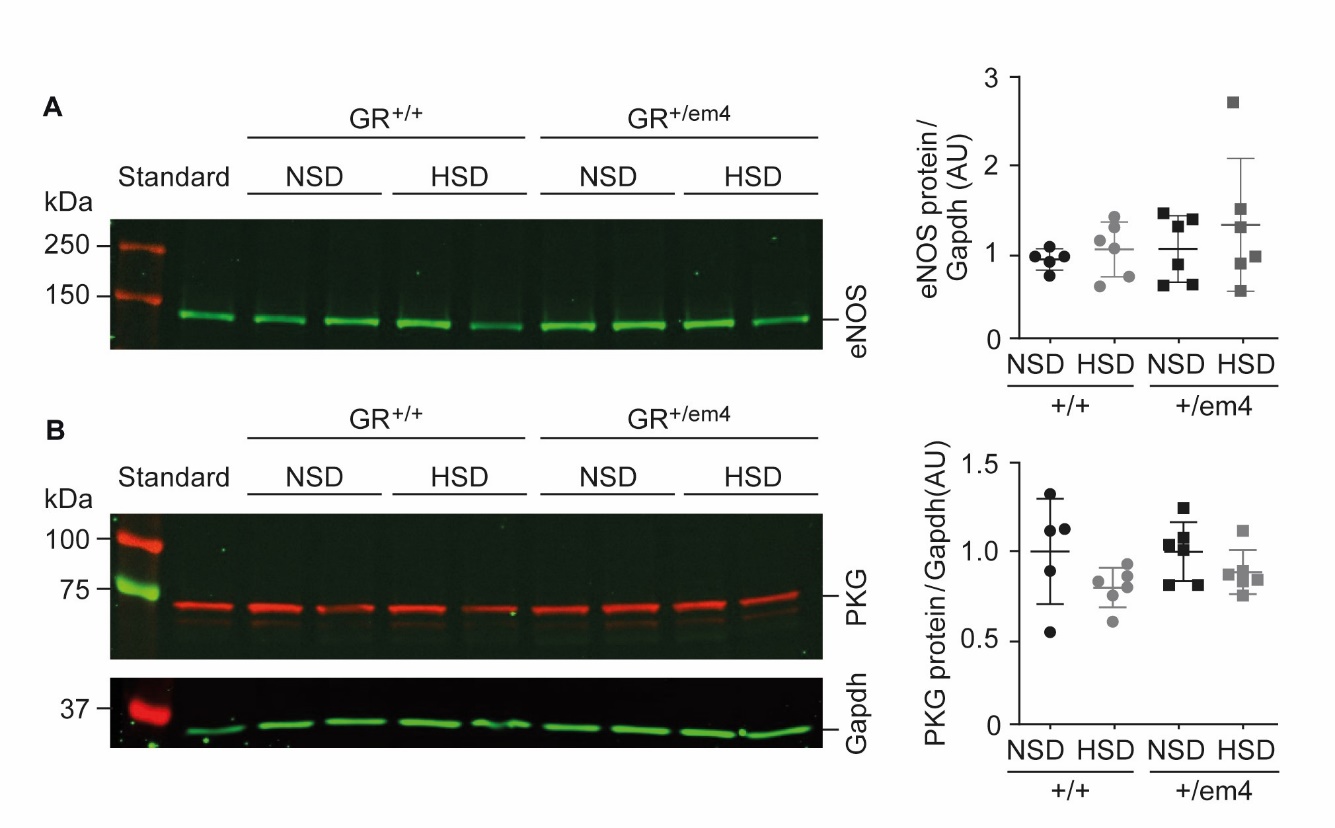


**eNOS and PKG protein abundances were similar in thoracic aortas of wildtype and GR^+/em4^ rats.**

Representative Western blot analyses of (**A**) endothelial nitric oxyde synthase (eNOS) and (**B**) protein-kinase G (PKG) of thoracic aortas from wildtype and GR^+/em4^ rats and their protein content quantifications. Data were analysed by unpaired two-tailed *t*-test with Welch correction using GraphPad Prism (GraphPad Software, version 9.0, Inc, San Diego, CA, USA) and values are indicated as mean ± SEM; NSD, black and HSD, grey circles and squares; n= 5-6 rats per group. Data were considered significant at *, *P* < 0.05.
